# Modern Technology in Multi-Shell Diffusion MRI Reveals Diffuse White Matter Changes in Young Adults With Relapsing-Remitting Multiple Sclerosis

**DOI:** 10.3389/fnins.2021.665017

**Published:** 2021-08-10

**Authors:** Ann-Marie Beaudoin, François Rheault, Guillaume Theaud, Frédéric Laberge, Kevin Whittingstall, Albert Lamontagne, Maxime Descoteaux

**Affiliations:** ^1^Department of Neurology, Faculty of Medicine and Health Sciences, Université de Sherbrooke, Sherbrooke, QC, Canada; ^2^Sherbrooke Connectivity Imaging Laboratory (SCIL), Université de Sherbrooke, Sherbrooke, QC, Canada; ^3^Department of Radiology, Faculty of Medicine and Health Sciences, Université de Sherbrooke, Sherbrooke, QC, Canada

**Keywords:** multiple sclerosis, high angular resolution imaging, free-water imaging, tractometry, cognition

## Abstract

**Objective:**

To characterize microstructural white matter changes related to relapsing-remitting multiple sclerosis using advanced diffusion MRI modeling and tractography. The association between imaging data and patient’s cognitive performance, fatigue severity and depressive symptoms is also explored.

**Methods:**

In this cross-sectional study, 24 relapsing-remitting multiple sclerosis patients and 11 healthy controls were compared using high angular resolution diffusion imaging (HARDI). The imaging method includes a multi-shell scheme, free water correction to obtain tissue-specific measurements, probabilistic tracking algorithm robust to crossing fibers and white matter lesions, automatic streamlines and bundle dissection and tract-profiling with tractometry. The neuropsychological evaluation included the Symbol Digit Modalities Test, Paced Auditory Serial Addition Test, Modified Fatigue Impact Scale and Beck Depression Inventory-II.

**Results:**

Bundle-wise analysis by tractometry revealed a difference between patients and controls for 11 of the 14 preselected white matter bundles. In patients, free water corrected fractional anisotropy was significantly reduced while radial and mean diffusivities were increased, consistent with diffuse demyelination. The fornix and left inferior fronto-occipital fasciculus exhibited a higher free water fraction. Eight bundles showed an increase in total apparent fiber density and four bundles had a higher number of fiber orientations, suggesting axonal swelling and increased organization complexity, respectively. In the association study, depressive symptoms were associated with diffusion abnormalities in the right superior longitudinal fasciculus.

**Conclusion:**

Tissue-specific diffusion measures showed abnormalities along multiple cerebral white matter bundles in patients with relapsing-remitting multiple sclerosis. The proposed methodology combines free-water imaging, advanced bundle dissection and tractometry, which is a novel approach to investigate cerebral pathology in multiple sclerosis. It opens a new window of use for HARDI-derived measures and free water corrected diffusion measures. Advanced diffusion MRI provides a better insight into cerebral white matter changes in relapsing-remitting multiple sclerosis, namely diffuse demyelination, edema and increased fiber density and complexity.

## Introduction

Multiple sclerosis (MS) is a neurodegenerative disease of the central nervous system characterized by chronic inflammation, demyelination, axonal degeneration, and gliosis (Compston and Coles, 2008). In addition to various physical signs and symptoms, affected individuals present variable degrees of cognitive impairment, which has a prevalence ranging from 40 to 70% in MS patients (Amato et al., 2006; Chiaravalloti and DeLuca, 2008; Langdon, 2011). Information processing speed, episodic memory and executive functions are frequently affected cognitive domains, even early in the disease (Rocca et al., 2015; Johnen et al., 2017). Comorbid depression (Feinstein, 2011) and fatigue (Chiaravalloti and DeLuca, 2008) are also highly prevalent in patients with MS. Fatigue is one of the most disabling symptoms and affects up to 80% of patients (Chaudhuri and Behan, 2004). The underlying cause of MS-related fatigue is still poorly understood but it is thought to have a central origin (Filippi et al., 2002; DeLuca et al., 2008).

Although conventional MRI is currently the mainstay of diagnosis and monitoring of disease activity in MS, it cannot capture the extent of MS-related cerebral damage. MRI-pathology correlation studies have shown abnormalities outside the typical MS lesions, extending in the “normal-appearing white matter” (NAWM) (Moore and Laule, 2012). Various pathological findings were discovered in the NAWM of MS patients, including diffuse inflammation and gliosis, demyelination, microglial activation (Allen et al., 2001; Kutzelnigg et al., 2005) and evidence of blood-brain barrier breakdown (Plumb et al., 2002). More advanced MRI approaches are needed to better investigate those changes in the NAWM. Moreover, the correlation between disability progression and white matter (WM) lesion burden is modest. This “clinico-radiological paradox” suggests the need for more advanced imaging techniques to monitor disease activity (Barkhof, 2002) and to better explain physical disability and cognitive impairment associated with MS, particularly in the early phase of the disease. The validation of diagnostic and prognostic biomarkers is also crucial for the development of new neuroprotective and repair treatments.

Diffusion MRI is increasingly used to investigate cerebral WM microstructural changes in MS and most of the previous studies used diffusion tensor imaging (DTI) derived measures. In relapsing-remitting multiple sclerosis (RRMS) patients, DTI studies showed a decreased fractional anisotropy (FA) and increased medial (MD), axial (AD) and radial (RD) diffusivities. These microstructural changes were correlated with increased disability and decreased neurocognitive performance (Zhang et al., 2016; Riccitelli et al., 2019; Tóth et al., 2019). Even if diffusion MRI has evolved in the past few years, DTI measures lack specificity to the different sub-components of the WM (Beaulieu, 2002). In fact, the various pathological processes occurring in MS cannot be differentiated since they have similar impact on the DTI tensor (Wang et al., 2011).

The goals of the present work are to first investigate cerebral WM changes in RRMS patients using modern technology in multi-shell diffusion MRI and second to explore the association between specific WM bundles abnormalities and the level of cognitive impairment, fatigue and depressive symptoms. The chosen methodology aims to overcome different well-known bias of the standard DTI method in an effort to be more specific to the underlying microstructure. The major components of the imaging protocol are fourfold: (i) advanced local modeling including fiber orientation distribution function (fODF) and free-water imaging, (ii) state-of-the-art probabilistic tractography using lesion-corrected WM mask, (iii) advanced WM bundling and (iv) cutting-edge bundle-wise statistics with tractometry. The combination of those technological elements and their application to RRMS patients is innovative, particularly the use of free-water imaging and tractometry.

## Materials and Methods

### Participants

We recruited 24 patients with RRMS (women/men = 20/4; mean age = 29, range = 22–35 years; mean disease duration = 4.3, range = 0.4–12 years) from the Neurology Clinic of the *Centre hospitalier universitaire de Sherbrooke* in Sherbrooke, Canada. Recruitment took place from September 2016 to March 2017 after obtaining approval of the institutional ethical committee (*Comité d’éthique de la recherche du CIUSSS de l’Estrie—CHUS*). Written informed consent was obtained according to the Declaration of Helsinki. To be included in the study, patients had to fit the following criteria: (1) diagnosis of RRMS from a neurologist after the age of 18 years (adult form of MS) with no sign of progression to secondary progressive MS at the time of recruitment (Polman et al., 2011); (2) no major vision or speech deficits; (3) no other known neurological disease; (4) no contraindication to MRI. Eleven subjects from the first time point of the *Penthera 3T* dataset (available on Zenodo: ^1^) were used for imaging comparison as a healthy control group (women/men = 3/8; mean age = 26, age range = 24–30 years). They were subjected to the same imaging protocol carried out on the same scanner as the RRMS group.

### Clinical Assessment

The same day as the MRI study, all patients underwent a neurological examination with rating using the Expanded Disability Status Scale (EDSS) to assess overall disability (Kurtzke, 1983). Paced Auditory Serial Addition Test, version 3 s (PASAT-3) was used to assess divided attention and working memory functions (Gronwall, 1977). The oral version of the Symbol-Digit Modalities Test (SDMT) (Smith, 1982) was used to assess information processing speed, visual working memory and sustained attention (Benedict et al., 2017). All patients were naive to those tests. To assess fatigue severity, a translated and adapted version of the Fatigue Impact Scale for French-speaking patients was completed (*Échelle modifiée d’impact de la fatique en sclérose en plaques* or EMIF-SEP) (Debouverie et al., 2009). To assess the severity of depressive symptoms, the Beck Depression Inventory-Second Edition (BDI-II) (Beck et al., 1996) was chosen.

### MRI Data Acquisition

Cerebral MRI was carried out on a 3T Philips Ingenia system. The average acquisition time was 30 min. T1-weighted, diffusion-weighted imaging and T2-fluid attenuation inversion recovery (T2-FLAIR) scans were used. T1-weighted (voxel resolution 1 mm × 1 mm × 1 mm), T2-FLAIR (voxel resolution 1 mm × 1 mm × 1 mm) and multi-shell diffusion-weighted imaging (8 *b* = 0 mm^2^/s, 8 *b* = 300 mm^2^/s, 32 *b* = 1,000 mm^2^/s, 60 *b* = 2,000 mm^2^/s for a total of 108 total diffusion volume; voxel resolution of 2 mm× 2 mm × 2 mm; TE/TR 95 ms/5,615 ms) were acquired for all participants. A reverse encoding b0 was acquired for distortion correction (Andersson et al., 2003).

### MRI Processing Pipeline

**FIGURE 1:**
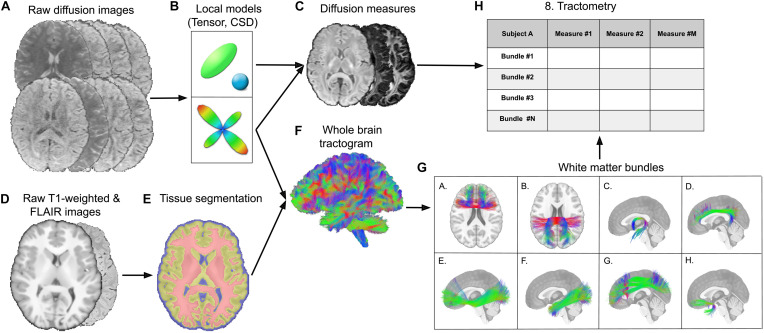
MRI processing pipeline. **(A)** Inputs diffusion-weighted images are processed by the *TractoFlow pipeline.* **(B)** Local modeling of the diffusion bi-tensor with tissue and free-water compartments. The fODF are estimated using constrained spherical deconvolution. **(C)** Diffusion MRI-derived measures and free-water fraction are computed. **(D)** Raw T1-weighted and T2-FLAIR images. **(E)** Manual and automatic lesions segmentations are performed in order to allow tracking through lesions. **(F)** Probabilistic tractography is computed in the lesion-corrected white matter mask. **(G)** Extraction of the preselected white matter bundles by *RecoBundles.* **(H)** Tractometry of each bundle using the previously computed diffusion measures and free-water fraction.

The processing pipeline is summarized in Figure 1. Diffusion-weighted images were processed using the *TractoFlow* pipeline (Di Tommaso et al., 2017; Kurtzer et al., 2017; Theaud et al., 2020), which is fully automated and has been proved to be highly reproducible in time and in immediate tes*t*-test (Theaud et al., 2020). It is publicly available in order to promote efficient, robust and reproducible diffusion tractography processing for open science. Free water corrected diffusion MRI measures (FAt, MDt and RDt, where “t” is for “tissue”) were computed from the *b* = 300 mm^2^/s and *b* = 1,000 mm^2^/s shells as well as the free water fraction (Pasternak et al., 2009). The free water elimination is not part of the *TractoFlow* pipeline and needs to be done separately. High angular resolution diffusion imaging (HARDI) was used to extract the principal directions of the fODF in order to make tractography more robust to crossing fibers. The fODF were estimated using constrained spherical deconvolution (Descoteaux et al., 2007; Tournier et al., 2007) from the *b* = 1,000 mm^2^/s and *b* = 2,000 mm^2^/s. HARDI-derived measures, total apparent fiber density (AFD_*tot*_) and number of fiber orientation (NuFO), were then extracted from the fODF.

**FIGURE 2:**
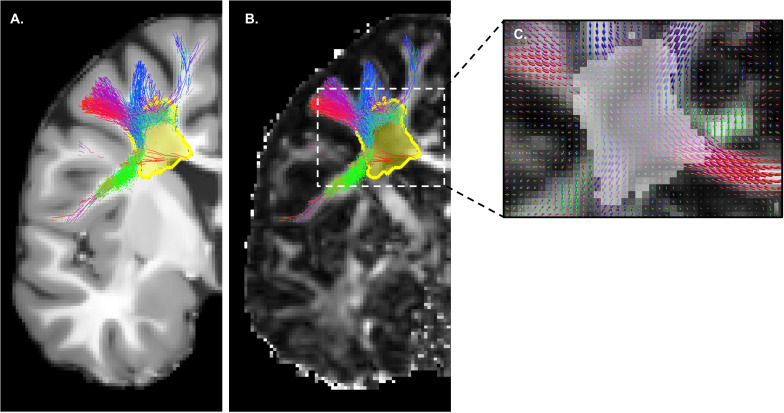
Tracking through white matter lesions. Manual and automatic segmentations are fused to produce the “lesion mask” (in yellow), covering all white matter lesions visible on the T2-FLAIR and T1-weighted **(A)** MRI images. Probabilistic tractography based on fODF peaks **(C)** is then executed and tracking is allowed through lesions, as shown on the FA map **(B)**. Without this correction, no track would be generated inside the yellow mask.

To ensure an adequate delineation of the WM lesions, both manual and automatic segmentation approaches were necessary. Manual delineation was performed using the T2-FLAIR and T1-weighted images. The automatic delineation was performed on the same images using a machine-learning based 3D U-net (Ronneberger et al., 2015). The manual and automatic segmentations were fused and voxels in either of the segmentations were considered as being part of the WM tissue. They were then added to the WM mask from the *FSL fast* tool (Woolrich et al., 2009) generated with the T1-weighted images, in order to allow tractography to track through lesions (Figure 2). Probabilistic tractography (Descoteaux et al., 2009; Tournier et al., 2012) was subsequently computed at 10 seeds per voxel in the lesion-corrected WM mask to achieve sufficient density and spatial coverage. The lesion-corrected WM mask was also used to estimate the fODF. A relative fODF amplitude threshold of 0.1 was set as a stopping criterion.

**FIGURE 3:**
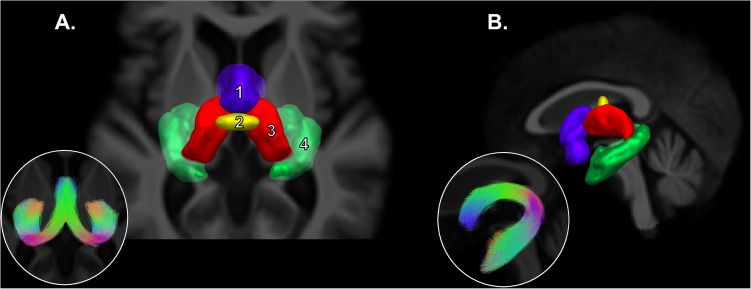
Fornix bundle-specific segmentation. Adapted from Rheault et al., 2018. Axial view **(A)** and sagittal view **(B)** of the regions of interest used for the manual segmentation of the fornix. Mamillary bodies in purple (inclusion, 1), body of the fornix in yellow (inclusion, 2), both thalami in red (exclusion, 3) and both hippocampi in green (inclusion, 4). In thumbnail, the general morphology of the segmented fornix is presented.

A multi-atlas and multi-parameter version of *RecoBundles* (Garyfallidis et al., 2018) was used to extract the preselected WM bundles. All previous tools were provided in the Dipy library (Garyfallidis et al., 2014). *RecoBundles* is an algorithm that recognizes bundles using a similarity metric between a subject’s streamline and a template or atlas. The utilized version (*RecobundlesX*) is an extension where the algorithm is executed multiple times, with variation in parameters, followed by label fusion. This tool is based on shape similarity to a template built from delineation rules inspired by anatomical priors from Catani and Thiebaut de Schotten, 2008. The template is available at: ^2^. A bundle-specific tractography approach was used for the fornix to reconstruct this “hard-to-track” pathway for both groups (Rheault et al., 2018, 2019). The manual segmentation of the fornix is based on a template of streamlines and anatomical priors that represent the shape, position and the endpoints of this bundle (Figure 3). Below the ventricles, tracking was allowed in the gray matter to maximize the chance of fully reconstructing the fornix despite the partial volume effect.

Once the bundle extraction was completed, visual quality assessment was made for each bundle in every subject to ensure the validity of the segmentation. The bundle volume was calculated for the whole bundle. The total lesion load was defined as the total volume of tissue included within the global lesion mask after manual and automatic segmentations. The lesion volume was also calculated for the 14 individual bundles. Subsequently, tractometry (Cousineau et al., 2017) of each bundle was performed using the previously computed diffusion measures and free water fraction. This method was chosen since, depending on the underlying WM fibers organization, the MRI-derived measures may vary along the studied bundles (Yeatman et al., 2012). Tractometry provides a higher sensitivity to the pathways’ microstructure by mapping a set of measures over the WM bundles. Finally, each bundle was separated along its length into five segments to provide further insight.

### Selection of the White Matter Bundles of Interest

**FIGURE 4:**
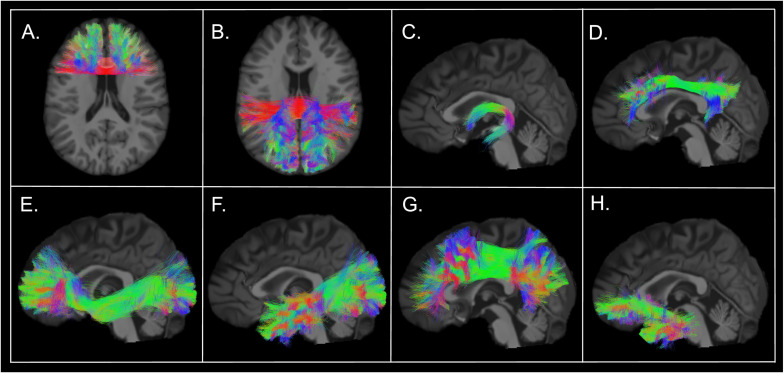
Selected white matter bundles of interest. White matter bundles obtained from the tractogram of a RRMS patient (female, 23 years old, disease duration of 5 years, EDSS 1.5, treated with Natalizumab). Extraction with *RecoBundles*, which uses bundle models as shape priors for detecting similar tracks and bundles in tractograms. Tracks segmented from the participant’s data were visually assessed to ensure quality of the bundle extraction. **(A)** Anterior corpus callosum (rostrum, genu); **(B)** Posterior corpus callosum (isthmus, splenium, tapetum); **(C)** Fornix; **(D)** Cingulum; **(E)** Inferior fronto-occipital fasciculus; **(F)** Inferior longitudinal fasciculus; **(G)** Superior longitudinal fasciculus; **(H)** Uncinate fasciculus.

According to the current knowledge regarding the function of the different WM fascicles of the human brain, corpus callosum (CC), cingulum, fornix, inferior fronto-occipital fasciculus (IFOF), superior longitudinal fasciculus (SLF), uncinate fasciculus (UF) and inferior longitudinal fasciculus (ILF) were selected as bundles of interest (Figure 4). Because of its role primarily in motor and sensitive functions, it was decided to exclude the body of the CC. Thus, the anterior and posterior CC were studied separately. The selection of a restricted number of WM bundles was also made to minimize the number of statistical tests and avoid false positive results.

### Statistical Analysis

All statistical tests were performed with *IBM SPSS Statistics 25* (SPSS Inc., Chicago, Illinois). The preselected WM bundles were studied separately. First, for each diffusion MRI measure (FAt, RDt, MDt, free water fraction, AFD_*tot*_, NuFO), the difference between RRMS patients and healthy controls was investigated with the appropriate parametric (independent samples *T*-test) and non-parametric (Mann–Whitney test) tests. The condition of a normal distribution was verified using Shapiro–Wilk test and visual assessment of the Q-Q plot. A unilateral *p* value < 0.01 was set for statistical significance after Bonferroni adjustment, considering each WM bundle separately. For more in-depth exploration, each bundle was then divided into five sections and the difference between the two groups was assessed for each of the bundle’s subsections. The purpose of this analysis was to verify that the observed changes were well distributed along the bundle since fanning of the fibers at the extremities of a bundle can bias diffusion measures.

When a significant difference between the two groups was found in the bundle as a whole, the association between neuropsychological testing results (SDMT, PASAT-3, EMIF-SEP, and BDI-II) and diffusion MRI measures was assessed with the Spearman’s rank correlation coefficient (*r*_*s*_) for RRMS patients. A *p* value of < 0.05 was determined to be statistically significant for the correlation analysis. Finally, the association between the neuropsychological assessment and the lesion load (global and for each bundle individually) was investigated.

## Results

### Clinical Assessment

Among the recruited RRMS patients, 12 patients were taking first line medication [Interferon beta-1a (2), Teriflunomide (5), Dimethyl fumarate (5)] and 11 were taking second line medication [Fingolimod (5), Natalizumab (5), Alemtuzumab (1)]. Only one patient was not taking any medication to treat MS. The median EDSS score was 1.5, with a range of 0 to 3. The SDMT mean score was 62.13 ± 8.13 and the PASAT-3 mean score was 49.00 ± 8.40. The mean score at the EMIF-SEP questionnaire was 83.96 ± 30.08. Every patient had a certain degree of fatigue, with 50% having a low level, 29% a moderate level and 21% a high level of fatigue. At the BDI-II questionnaire, the mean score was 11.13 ± 10.85 with 67% of patients having a normal score while 21% had a score indicating mild depression. We identified three cases (12%) with scores consistent with a diagnosis of severe depression and they were referred to the appropriate health professionals.

### MRI Findings

In the RRMS group, the average whole brain lesion volume was 9.09 ± 7.08 mL. Table 1 shows the lesion volume of the 14 selected WM bundles. A positive association was established between the free water fraction and the proportion of lesioned tissue of the anterior CC (*r*_*s*_ = 0.523, *p* = 0.009), posterior CC (*r*_*s*_ = 0.578, *p* = 0.003) bilateral IFOF (*r*_*s*_ = 0.584, *p* = 0.003 for the right side and *r*_*s*_ = 0.694, *p* < 0.001 for the left side), ILF (*r*_*s*_ = 0.774, *p* < 0.001 for the right side and *r*_*s*_ = 0.775, *p* < 0.001 for the left side) and SLF (*r*_*s*_ = 0.753, *p* < 0.001 for the right side and *r*_*s*_ = 0.705, *p* < 0.001 for the left side).

**TABLE 1:** Lesion volume per bundle in relapsing-remitting multiple sclerosis patients.

**WM Bundles**	**T2 lesion volume (Mean ± SD; mL)**	**Proportion of lesioned tissue per bundle (Mean ± SD)**	**Association between T2 lesion volume and free water fraction**
Anterior CC	4.01 ± 2.94	0.026 ± 0.019	*r*_*s*_ = 0.523/*p* = 0.009*
Posterior CC	5.95 ± 4.41	0.040 ± 0.030	*r*_*s*_ = 0.578/*p* = 0.003*
Right cingulum	0.64 ± 0.61	0.034 ± 0.031	*r*_*s*_ = −0.009/*p* = 0.968
Left cingulum	0.67 ± 0.69	0.030 ± 0.028	*r*_*s*_ = 0.348/*p* = 0.096
Right fornix	0.24 ± 0.30	0.025 ± 0.033	*r*_*s*_ = 0.331/*p* = 0.114
Left fornix	0.20 ± 0.20	0.022 ± 0.022	*r*_*s*_ = 0.359/*p* = 0.085
Right IFOF	3.15 ± 2.01	0.043 ± 0.029	*r*_*s*_ = 0.584/*p* = 0.003*
Left IFOF	2.67 ± 1.74	0.035 ± 0.022	*r*_*s*_ = 0.694/*p* < 0.001*
*R*ight ILF	2.85 ± 2.29	0.038 ± 0.031	*r*_*s*_ = 0.774/*p* < 0.001*
Left ILF	2.25 ± 1.61	0.031 ± 0.022	*r*_*s*_ = 0.775/*p* < 0.001*
Right SLF	2.63 ± 2.75	0.031 ± 0.033	*r*_*s*_ = 0.753/*p* < 0.001*
Left SLF	2.16 ± 1.88	0.025 ± 0.023	*r*_*s*_ = 0.705/*p* < 0.001*
Right UF	0.77 ± 0.50	0.023 ± 0.015	*r*_*s*_ = 0.487/*p* = 0.016
Left UF	0.70 ± 0.42	0.024 ± 0.013	*r*_*s*_ = 0.286/*p* = 0.175

CC, corpus callosum; IFOF, inferior fronto-occipital fasciculus; ILF, inferior longitudinal fasciculus; r_*s*_, Spearman’s rank correlation coefficient; SD, standard deviation; SLF, superior longitudinal fasciculus; UF, uncinate fasciculus; WM, white matter.

* statistically significant difference (*p* < 0.01).

As shown in Table 2, differences in free water corrected measures (FAt, RDt, MDt) were established between RRMS patients and the control group for all the selected WM bundles except both sides of the fornix and left cingulum. Values of FAt were reduced in the RRMS group, while RDt and MDt were increased. The fornix and left IFOF showed a significant increase in free water fraction. For the other bundles, the values were constantly higher in the RRMS group, but the difference did not reach statistical significance. AFD_*tot*_ was significantly increased in the anterior CC, left cingulum, left IFOF, right and left ILF, left SLF and right and left UF of the recruited patients. NuFO was also increased in the anterior CC, right and left IFOF and left UF of the RRMS group. The subsequent analysis demonstrated that the observed differences were well distributed along the five subsections of each bundle (data not shown).

**TABLE 2:** Differences in diffusion MRI measures between multiple sclerosis patients and healthy controls.

**Bundle**	**Groups**	**FAt (Mean ± SD)**	**RDt (Mean ± SD)**	**MDt (Mean ± SD)**	**Free water fraction (Mean ± SD)**	**AFD_*tot*_ (Mean ± SD)**	**NuFO (Mean ± SD)**
Anterior CC	RRMS	0.640 ± 0.017*	46 × 10^–5^ ± 2.2 × 10^–5^*	81 × 10^–5^ ± 1.5 × 10^–5^*	0.056 ± 0.017	0.307 ± 0.019*	1.152 ± 0.131*
	HC	0.665 ± 0.023	43 × 10^–5^ ± 2.8 × 10^–5^	78 × 10^–5^ ± 1.9 × 10^–5^	0.049 ± 0.007	0.289 ± 0.011	1.044 ± 0.061
Posterior CC	RRMS	0.685 ± 0.017*	40 × 10^–5^ ± 2.1 × 10^–5^*	77 × 10^–5^ ± 1.4 × 10^–5^*	0.070 ± 0.017	0.341 ± 0.015	1.246 ± 0.090
	HC	0.703 ± 0.014	38 × 10^–5^ ± 1.7 × 10^–5^	75 × 10^–5^ ± 1.2 × 10^–5^	0.055 ± 0.010	0.327 ± 0.011	1.206 ± 0.076
Right cingulum	RRMS	0.637 ± 0.025*	46 × 10^–5^ ± 3.1 × 10^–5^*	81 × 10^–5^ ± 2.0 × 10^–5^*	0.028 ± 0.006	0.299 ± 0.014	1.204 ± 0.136
	HC	0.679 ± 0.017	41 × 10^–5^ ± 2.1 × 10^–5^	77 × 10^–5^ ± 1.4 × 10^–5^	0.023 ± 0.007	0.292 ± 0.008	1.450 ± 0.069
Left cingulum	RRMS	0.667 ± 0.022	43 × 10^–5^ ± 2.7 × 10^–5^	78 × 10^–5^ ± 1.8 × 10^–5^	0.041 ± 0.009	0.298 ± 0.014*	1.183 ± 0.117
	HC	0.684 ± 0.019	40 × 10^–5^ ± 2.4 × 10^–5^	77 × 10^–5^ ± 1.6 × 10^–5^	0.032 ± 0.009	0.284 ± 0.010	1.111 ± 0.076
Right fornix	RRMS	0.709 ± 0.020	35 × 10^–5^ ± 2.7 × 10^–5^	73 × 10^–5^ ± 2.0 × 10^–5^	0.482 ± 0.066*	0.272 ± 0.026	1.067 ± 0.101
	HC	0.705 ± 0.018	36 × 10^–5^ ± 2.6 × 10^–5^	74 × 10^–5^ ± 1.9 × 10^–5^	0.415 ± 0.046	0.274 ± 0.014	1.014 ± 0.104
Left fornix	RRMS	0.698 ± 0.027	37 × 10^–5^ ± 4.0 × 10^–5^	74 × 10^–5^ ± 3.0 × 10^–5^	0.465 ± 0.091*	0.264 ± 0.028	1.019 ± 0.099
	HC	0.696 ± 0.020	38 × 10^–5^ ± 2.6 × 10^–5^	75 × 10^–5^ ± 1.8 × 10^–5^	0.384 ± 0.034	0.269 ± 0.012	0.982 ± 0.104
Right IFOF	RRMS	0.608 ± 0.021*	50 × 10^–5^ ± 2.5 × 10^–5^*	83 × 10^–5^ ± 1.7 × 10^–5^*	0.065 ± 0.018	0.309 ± 0.011	1.176 ± 0.112*
	HC	0.642 ± 0.017	45 × 10^–5^ ± 2.1 × 10^–5^	80 × 10^–5^ ± 1.4 × 10^–5^	0.052 ± 0.011	0.297 ± 0.016	1.094 ± 0.059
Left IFOF	RRMS	0.606 ± 0.020*	50 × 10^–5^ ± 2.5 × 10^–5^*	83 × 10^–5^ ± 1.7 × 10^–5^*	0.059 ± 0.011*	0.307 ± 0.013*	1.178 ± 0.113*
	HC	0.645 ± 0.021	45 × 10^–5^ ± 2.5 × 10^–5^	80 × 10^–5^ ± 1.7 × 10^–5^	0.048 ± 0.010	0.291 ± 0.014	1.075 ± 0.060
Right ILF	RRMS	0.599 ± 0.019*	51 × 10^–5^ ± 2.4 × 10^–5^*	84 × 10^–5^ ± 1.6 × 10^–5^*	0.061 ± 0.019	0.305 ± 0.011*	1.205 ± 0.134
	HC	0.630 ± 0.018	47 × 10^–5^ ± 2.3 × 10^–5^	81 × 10^–5^ ± 1.5 × 10^–5^	0.049 ± 0.010	0.291 ± 0.015	1.090 ± 0.084
Left ILF	RRMS	0.597 ± 0.018*	51 × 10^–5^ ± 2.3 × 10^–5^*	84 × 10^–5^ ± 1.5 × 10^–5^*	0.059 ± 0.014	0.305 ± 0.011*	1.211 ± 0.147
	HC	0.630 ± 0.019	47 × 10^–5^ ± 2.4 × 10^–5^	81 × 10^–5^ ± 1.6 × 10^–5^	0.047 ± 0.010	0.287 ± 0.016	1.085 ± 0.087
Right SLF	RRMS	0.607 ± 0.024*	50 × 10^–5^ ± 2.9 × 10^–5^*	83 × 10^–5^ ± 1.9 × 10^–5^*	0.010 ± 0.005	0.323 ± 0.014	1.433 ± 0.173
	HC	0.637 ± 0.023	46 × 10^–5^ ± 2.8 × 10^–5^	81 × 10^–5^ ± 1.9 × 10^–5^	0.008 ± 0.003	0.313 ± 0.012	1.356 ± 0.111
Left SLF	RRMS	0.597 ± 0.021*	51 × 10^–5^ ± 2.6 × 10^–5^*	84 × 10^–5^ ± 1.7 × 10^–5^*	0.011 ± 0.006	0.331 ± 0.015*	1.471 ± 0.167
	HC	0.620 ± 0.015	48 × 10^–5^ ± 1.9 × 10^–5^	82 × 10^–5^ ± 1.3 × 10^–5^	0.008 ± 0.003	0.316 ± 0.012	1.378 ± 0.134
Right UF	RRMS	0.554 ± 0.023*	56 × 10^–5^ ± 2.9 × 10^–5^*	88 × 10^–5^ ± 1.9 × 10^–5^*	0.030 ± 0.017	0.287 ± 0.010*	1.090 ± 0.166
	HC	0.580 ± 0.028	53 × 10^–5^ ± 3.5 × 10^–5^	85 × 10^–5^ ± 2.3 × 10^–5^	0.023 ± 0.007	0.273 ± 0.016	0.949 ± 0.111
Left UF	RRMS	0.543 ± 0.020*	58 × 10^–5^ ± 2.5 × 10^–5^*	89 × 10^–5^ ± 1.7 × 10^–5^*	0.029 ± 0.018	0.286 ± 0.014*	1.107 ± 0.176*
	HC	0.569 ± 0.024	54 × 10^–5^ ± 2.9 × 10^–5^	86 × 10^–5^ ± 2.0 × 10^–5^	0.019 ± 0.006	0.271 ± 0.012	0.954 ± 0.095

AFDtot, total apparent fiber density; CC, corpus callosum; FAt, tissue fractional anisotropy; HC, healthy controls; IFOF, inferior fronto-occipital fasciculus; ILF, inferior longitudinal fasciculus; MDt (mm/s^2^), tissue mean diffusivity; NuFO, number of fiber orientation; RDt (mm/s^2^), tissue radial diffusivity; RRMS, relapsing-remitting multiple sclerosis; SD, standard deviation; SLF, superior longitudinal fasciculus; UF, uncinated fasciculus.

*statistically significant difference (*p* < 0.01).

### Correlation Between Imaging and Clinical Data

The free water corrected diffusion MRI measures along the right SLF showed an association with the BDI-II questionnaire results. A decrease in FAt (*r*_*s*_ = −0.476, *p* = 0.019), an increase in RDt (*r*_*s*_ = 0.476, *p* = 0.019) and an increase in MDt (*r*_*s*_ = 0.468, *p* = 0.021) were associated with a higher level of depressive symptoms. Free water fraction and HARDI-derived measures (AFD_*tot*_, NuFO) were not associated with the clinical results. Furthermore, no correlation was found between the neurocognitive testing results and the global brain lesion load. When the WM bundles were studied individually, their respective proportion of lesioned tissue was also not associated with the clinical data.

## Discussion

The purpose of the present study was to use advanced diffusion MRI modeling and tractography to characterize RRMS-related WM changes. To our knowledge, this study is the first to examine cerebral WM changes using advanced local modeling including fODF and free water correction in the context of MS. Also, it is the first study to use tractometry to investigate the association between diffusion MRI-derived measures and neuropsychological symptoms of MS. At every step, from MRI data acquisition to statistical analysis, choices were made in an effort to obtain measures that are more specific to the pathological processes occurring in MS.

### Advantages of Modern Technology in Diffusion MRI

To begin with, it has been shown that multi-shell diffusion MRI provides a better estimation of the free water corrected measures and free water fraction than single-shell data (Pasternak et al., 2012). Multi-shell acquisitions were also shown to improve the angular resolution of orientation distribution functions (Jeurissen et al., 2014). Moreover, HARDI was developed to provide new anisotropy measures (Tournier et al., 2011) and to solve the *crossing fiber problem* using multiple angular measurements to recover crossing configurations. This is crucial since the most important limitation of the DTI methodology is that, at every voxel, it can only model a single-fiber population. This represents a problem when imaging voxels contain multiple fiber populations like crossing, fanning and highly curving fibers. It has been estimated that between 66% and 90% of WM voxels contain crossing fibers (Jeurissen et al., 2013). This problem also leads to bias in DTI measures since several biological tissue abnormalities may lead to the same change in FA value or diffusivities, including myelin damage, axonal loss and crossing fibers deterioration (Alexander et al., 2007). HARDI enables the extraction of the principal directions of the fODF and this information makes tractography more robust.

With multi-shell diffusion MRI data, different techniques can generate biomarkers that are more specific than DTI to microstructural alterations in cerebral WM. Among those advanced diffusion MRI techniques, neurite orientation dispersion and density imaging (NODDI) is reported to be more sensitive for detection of MS lesions than conventional DTI (Zhang et al., 2012). NODDI separates the signal arising from three different tissue compartments (intraneurite water, extraneurite water and cerebrospinal fluid) to estimate neurite density and neurite orientation dispersion. These factors contribute to FA and analyzing them separately is an advantage. A recent study has shown a decrease in neurite density index in cerebral NAWM and spinal cord WM of RRMS patients (Collorone et al., 2020). This multi-compartment model requires acquisition time similar to our methodology (Zhang et al., 2012). It is important to note that NODDI is not robust to crossing fibers and one would still need to use constrained spherical deconvolution or another technique robust to crossing fibers to perform tractography. It was also shown that NODDI fails to consistently extract discrete measures of the numbers and orientations of fODF peaks (Schilling et al., 2018). DIstribution of 3D Anisotropic MicrOstructural eNvironments in Diffusion-compartment (DIAMOND) imaging (Scherrer et al., 2016) could also have been used to solve fiber crossings. This is a hybrid model accounting for several discrete compartments free isotropic attributed to CSF, restricted isotropic attributed to water in glial cells and water in and around WM bundles.

When an image voxel contains both brain tissue and free water such as edema, the DTI measures no longer represent the underlying tissue properties, and the observed changes can be caused by partial volume effect. To address this issue, free water elimination and mapping from diffusion MRI has been proposed (Pasternak et al., 2009). Free water elimination fits, for a single voxel, a bi-tensor model including a tissue part and a free water part. The assessment of extracellular water is important as it can bias diffusion measures by decreasing FA and increasing MD. It has been demonstrated that free water corrected measures have a higher sensitivity than conventional diffusion MRI measures and thus are considered to provide more tissue-specific measurements (Albi et al., 2017).

It is known that WM lesions can affect the tractography algorithm, track reconstruction and track-dissection (Lipp et al., 2020). Strategies must be employed to avoid premature track termination, as both FA threshold and T1-based segmentations would result in “holes” were tracking would not be possible. In the present study, the strategy was to fill the tracking mask with the use of a lesion-corrected WM segmentation mask combined with probabilistic tractography based on fODF amplitudes. If the diffusion MRI acquisition suggested a strong and coherent fODF peak, tracking used it even if it was inside a WM lesion. This method allows the reconstruction of tracks outside and inside MS lesions and thus increases anatomical accuracy.

Another important pitfall to consider is the high probability of false-positive tracks and bundles (Maier-Hein et al., 2017). To reduce the risk of false positive results, a restricted number of WM bundles were studied instead of doing a whole brain analysis. We selected WM fascicles that, when damaged, are associated with cognitive dysfunction according to the literature. To extract the WM bundles, the virtual dissection tool *RecoBundles* was chosen over manual segmentation because the latter has the disadvantage to be time consuming and can be bias by intra-rater and inter-rater variability (Rheault et al., 2020). *RecoBundles* is an advanced bundling tool proved robust to pathological brains (Garyfallidis et al., 2018). Visual inspection confirmed that the WM bundles reconstruction was successful and anatomically accurate for every participant despite the presence of WM lesions.

The fornix is an exception to this segmentation method since its anatomy makes it particularly difficult to reconstruct. The fornix is a very small and highly curving bundle in close proximity to the lateral ventricles. The partial volume effect with the cerebrospinal fluid is a major problem, as demonstrated in a recent DTI study in which only 36% of the participants had the full extent of the fornix recovered (Valdés Cabrera et al., 2020). According to the authors, the tractography transections were due to partial volume with cerebrospinal fluid lowering FA below the threshold for tractography (0.2) in the crus as it passes through the lateral ventricles. To overcome this limitation, a bundle-specific tractography approach was performed, using a template that better represent the shape and position of the fornix (Rheault et al., 2019). Both sides of the fornix were reconstructed for all participants, even in the patients with a higher lesion load. Free water corrected measures seem to be differentially affected in the fornix, showing no differences between RRMS patients and healthy controls. In the RRMS group, the free water fraction was significantly increased in this bundle and it might be caused by cerebrospinal fluid contamination.

Finally, tractometry was achieved by projecting the previously computed free water corrected measures and HARDI-derived measures on the segmented WM bundles. Most of previously published studies are based on voxel-wise analysis. This approach assumes that subjects in the same group have a similar brain configuration. If a difference is observed between a group of healthy subjects and a group of subjects with a neurological disease, it will be concluded that this difference is caused by the disease. However, virtually segmented WM bundles can be affected by noise and distortion to some level, which can bias the results. By producing streamlines-based statistics, tractometry was shown to have satisfactory reproducibility in both healthy subjects and individuals with Parkinson’s disease (Cousineau et al., 2017). A recent study utilized tract-specific MRI measures to investigate the relationship between neuropathology and cognition in MS (Winter et al., 2021). In this study, 40 patients with long-lasting MS were recruited. They had relatively low levels of disability and had never received disease modifying treatments. The imaging protocol included HARDI with multi-shell acquisition. The selected imaging measures included FA, MD, RD, AFD, NuFO, and rotationally invariant spherical harmonics (Mirzaalian et al., 2016). They also included white matter measures including T2 lesion load, whole brain tractogram load and bundle load. Finally, a lesion-specific approach to the tractometry framework (called lesionometry) was used to obtain measures specific to the streamlines traversing a lesion. Various cognitive measures were correlated to these imaging measures revealing that tract-specific measures outperformed the global cerebral lesion load and the tractogram load measures. This study has some similarities with our work regarding imaging methodology. One of the main differences is the use of free-water imaging. Lesionometry, as presented by Winter et al., is a new application of tractometry and it seems promising for MS neuropathology evaluation. In future work, a combination of both methodologies could be useful to better investigate the correlation between WM abnormalities and MS symptomatology.

### Application to Relapsing-Remitting Multiple Sclerosis

The pathological hallmark of RRMS is focal demyelinating lesions, but the damage has been proven to extend in the normal-appearing WM early in the disease course (De Santis et al., 2019). Using modern methodology including free-water imaging and tractometry, diffusion MRI-derived measures revealed a significant difference between RRMS patients and healthy controls for 11 of the 14 selected WM bundles, highlighting a diffuse pathological process. Significant decrease of FAt and increase of RDt and MDt were observed, suggestive of demyelination. It confirms prior work using standard DTI measures (De Santis et al., 2019; Lipp et al., 2019) and it’s in line with the current knowledge of RRMS pathology (Kutzelnigg et al., 2005). Moreover, the observed changes were well distributed along the bundles, increasing our confidence in the validity of the results. It’s important to mention that MS pathology is associated with inflammatory cells infiltration, including macrophages and lymphocytes. Astrocytes are also implicated in the formation and evolution of MS lesions (Ponath et al., 2018). The presence of astrocytic and microglial infiltration was associated with greater hindered diffusion in the extra-neurite space (Yi et al., 2019). The use of multi-compartment models in diffusion MRI, including a “glial cells compartment,” is promising to assess neuroinflammation association with microglial activation.

Values of free water fraction in the RRMS group were persistently higher across all studied bundles. The absence of significant difference in the bundles other than fornix and left IFOF may be explained by a lack of statistical power since the sample size was small. In addition, the majority of patients were under an immunomodulatory treatment at the time of the study and their anti-inflammatory effect may have limited the free water fraction increase. Interestingly, the lesion volume of most WM bundles was strongly associated with their respective free water fraction, showing the important contribution of the lesioned tissue in the estimated volume of free water. In addition, it has been shown that perivascular spaces are enlarged in MS (Granberg et al., 2020) and perivascular space fluid contributes to DTI changes in cerebral WM (Sepehrband et al., 2019). Water in perivascular space could therefore impact free water fraction values in MS. In a neurodegenerative disease like MS, this new parameter could be a useful indicator of inflammation and edema.

HARDI-derived measures also provided valuable information about WM microstructure. AFD_*tot*_ is a measure proportional to the underlying fiber density (Raffelt et al., 2012), and it was increased in the RRMS group. That finding is consistent with previous studies and may reflect axonal swelling caused by inflammation. A diffusion MRI study found that RRMS was associated with a widespread increase in axonal diameter (De Santis et al., 2019). Another recent study detected blister-like swellings of the axon-myelin unit in MS normal-appearing WM (Luchicchi et al., 2021). Microstructural axonal changes have also been observed in other neurodegenerative diseases. In a DTI study using a mouse model of Huntington’s disease, histological analysis of the corpus callosum showed axonal reorganization and increased tortuosity, myelin content reduction and astrogliosis (Gatto et al., 2019). Moreover, a diffusion MRI study investigated tract-specific differences at a within-voxel level (fixel-based analysis) in Alzheimer’s disease patients. It revealed reductions in apparent fiber density and bundle cross-section within specific white matter structures, suggesting substantial axonal loss associated with this disease (Mito et al., 2018). The fact that AFD_*tot*_ was not reduced in RRMS patients could be an indicator that axonal loss was not significant at this stage of the disease. On the other hand, NuFO was increased in some WM bundles in RRMS patients. NuFO represents the number of local peaks of the fiber orientation distribution in each voxel. This measure provides information about the complexity of the underlying WM organization. It has been proven to be highly consistent across individuals and thus could be a sensitive marker of disease-related changes (Dell’Acqua et al., 2013). This finding could be the result of compensatory neuroplasticity and neuronal reorganization mechanisms associated with RRMS (Fleischer et al., 2019).

### Association Between Clinical Data and Advanced Diffusion MRI Measures

A bundle-wise approach with tractometry was chosen to investigate the role of specific WM bundles in cognitive dysfunction, fatigue and depression associated with RRMS. The only association found was that a higher level of depressive symptoms was related to diffusion abnormalities along the right SLF. However, the role of this fasciculus is not clear in the setting of mood disorders. The association study was probably limited by the small sample size, causing a lack of statistical power. In addition, the recruited patients were found to have a relatively low level of impairment. They performed quite well during the cognitive testing and the chosen tests may be not sensitive enough in a young population of RRMS patients. Further studies with more subjects and a more thorough neurocognitive evaluation are necessary. Moreover, the brain can adapt to changes in its environment, for example when subjected to chronic inflammation. Cortical network reorganization and neuroplasticity could therefore explain the lack of association between neurocognitive deficits and diffusion MRI changes at the early stage of the disease (Fleischer et al., 2019).

The role of myelin damage in MS-related cognitive dysfunction has been demonstrated by other imaging techniques, like myelin water imaging (Abel et al., 2020), but there’s increasing evidence that gray matter damage plays a major role. Studies using DTI and conventional MRI measures showed that gray matter atrophy of various cortical and subcortical regions was associated with a reduction in PASAT performance (Uher et al., 2014; Riccitelli et al., 2019). Other imaging methods can also provide useful information regarding gray matter integrity and reorganization in MS. Cortical lesions can be assessed by ultra-high field imaging (Kilsdonk et al., 2016) and by double inversion recovery sequences (Seewann et al., 2012). Global and regional cortical atrophy can be captured by algorithms like SIENA (Smith et al., 2001) and FreeSurfer (Fischl, 2012). However, in patients with RRMS early in the disease course, morphometric measures might not be sensitive enough. Indeed, a longitudinal cortical network analysis revealed an increase in local and modular connections over a 12-month period in RRMS patients, in the absence of measurable cortical atrophy (Fleischer et al., 2019). Finally, MS patients exhibit cerebral functional connectivity abnormalities, including altered connectivity in deep-gray matter regions (Tahedl et al., 2018). Multi-modality imaging approaches would improve our understanding of this disease and its impact on cognition.

Global lesion load and lesion volume per bundle were not associated with the neuropsychological evaluation results. This might be explained by the location of the cerebral WM lesions in the brain of the studied patients. In MS, the importance of lesion location has been demonstrated (Kincses et al., 2011). Certain lesions are said to be “silent” because they are not associated with clinical signs or symptoms. Symptomatic lesions are usually located in eloquent WM areas and thus have a higher yield to cause neurological and functional impairment. In addition, T2 lesion load evaluation underestimates the damage to the NAWM and to the cortical and subcortical gray matter (Barkhof, 2002).

### Study Limitations

The primary limitation of the study is the small and heterogenous sample size, which limits results’ generalizability. Even if it reflects the reality in clinical practice, treatment heterogeneity is a limitation since disease-modifying therapies don’t have the same effectiveness in treating neuroinflammation. In a future study with a larger sample size, it would be interesting to divide patients into subgroups according to their treatment regimen (no treatment, first-line treatments and second-line treatments) to compare those more homogeneous samples with advanced diffusion MRI. In addition, the RRMS group is skewed toward females while the healthy control group comprise more men. This imbalance between the groups is firstly due to recruitment difficulties, mainly for men in our center. Also, the healthy control group was part of a pre-existing dataset (*Penthera 3T*) which mostly comprised men. Due to limited resources at the time of the study, the use of that cohort was more cost-effective. In future studies, it would be recommended that subjects be matched for age and sex.

Another limitation is the absence of comparison of diffusion MRI-derived measures inside the lesions and in the NAWM. It is thus impossible to determine if the increase in free water fraction for example is driven by the presence of focal lesions only. In future work, the use of intralesional measures and lesionometry (Winter et al., 2021) would be helpful to better characterize diffusion MRI abnormalities caused specifically by MS focal lesions. It would also be interesting to separate gadolinium-enhancing lesions (considered as “active lesions”) from non-enhancing lesions.

With regard to MRI data processing, fODF reconstruction may be affected by the free water part of the signal since free-water modeling is done separately from the fODF modeling. Therefore, AFD_*tot*_ and NuFO values may still be influenced by tissue changes that contribute to the observed increase in free water fraction. To limit this contamination, only higher b-values (*b* = 1,000 and *b* = 2,000) were used in the fitting and fODF reconstruction. How is constrained spherical deconvolution fODF reconstruction affected by free water is to the best of our knowledge still an open question. It could be argued that constrained spherical deconvolution and using higher b-values results in removing most of the isotropic diffusion part of the signal, but this has never been thoroughly studied. In future studies, joint modeling could be utilized to obtain AFD_*tot*_ and NuFO measures that are independent of free water contamination.

## Conclusion

We used a novel methodology to characterize cerebral WM in young patients with RRMS. The association between cognitive dysfunction, fatigue and depression and free water corrected diffusion measures and HARDI-derived measures was also studied for the first time. At the era of “disease modifying therapies,” it is crucial to develop more specific measures to disentangle WM microstructural changes caused by this disease and its treatment. To be able to probe the WM changes with advanced diffusion MRI, in combination with myelin-specific MRI contrasts such as myelin water imaging or inhomogeneous magnetization transfer, would be a great advantage for clinical research and this is part of future work.

## Data Availability Statement

The anonymized research data are available to the scientific community on request from the first author. Codes used for this study are available online: https://github.com/scilus/scilpy?fbclid=IwAR2xgpYuNW2Q6CoC06-6FnlLNVqu8SnIa6KzLS1QrBt4ztcMgLUoL3mLOf4. The multi-atlas bundle segmentation is available on Zenodo: https://zenodo.org/record/3613688?fbclid=IwAR0SNxO8LVgkYYAgKtfg9ZeshbfTvyXMeggqHnoRou_a2MmAaQsvxhroeC0#.YQybNxNKjPh. The control group dataset (Penthera 3T) is available on Zenodo: https://zenodo.org/record/2602049#.XXeZf5NKjy8.

## Ethics Statement

The studies involving human participants were reviewed and approved by Comité d’éthique de la recherche du CIUSSS de l’Estrie*—*CHUS. The patients/participants provided their written informed consent to participate in this study.

## Author Contributions

A-MB and MD directed the design and operation of the project. A-MB acquired the clinical and imaging data, conducted the data analyses, and led the manuscript preparation. AL was involved in the recruitment and clinical evaluation of the multiple sclerosis patients. FR processed the imaging data and participated in the creation of the figures. FL helped with the statistical analysis. All authors helped with the interpretation of the data and contributed to the reviewing and editing of the earlier versions of the manuscript.

## Conflict of Interest

The authors declare that the research was conducted in the absence of any commercial or financial relationships that could be construed as a potential conflict of interest.

## Publisher’s Note

All claims expressed in this article are solely those of the authors and do not necessarily represent those of their affiliated organizations, or those of the publisher, the editors and the reviewers. Any product that may be evaluated in this article, or claim that may be made by its manufacturer, is not guaranteed or endorsed by the publisher.

## Footnotes

1
https://zenodo.org/record/2602049#.XXeZf5NKjy8

2
https://zenodo.org/record/4104300#.YNoP1XVKiiM
